# Uremic and Post-Transplant Gastropathy in Patients With Chronic Kidney Disease and End-Stage Renal Disease

**DOI:** 10.7759/cureus.10578

**Published:** 2020-09-21

**Authors:** Alla Turshudzhyan, David Inyangetor

**Affiliations:** 1 Internal Medicine, University of Connecticut, Farmington, USA

**Keywords:** uremic gastropathy, renal transplant, mycophenolate mofetil, helicobacter pylori

## Abstract

Gastrointestinal (GI) mucosal lesions are common in chronic kidney disease (CKD), end-stage renal disease (ESRD), and in post-renal transplant period. However, etiology of mucosal lesions pre- and post-transplant is very different. Gastropathy in non-transplant ESRD patients usually develops because of uremia, chronic anemia, and fluctuations in the gastric blood supply during hemodialysis, eventually leading to uremic gastritis. Gastropathy in post-transplant patients tends to be associated with immunosuppressive therapies.

Helicobacter pylori infection is more prevalent in uremic patients than in post-transplant patients. Uremia can also lead to uremic arteriolopathy and autonomic nervous system dysfunction, which can present with GI symptoms mimicking uremic gastropathy. Post-transplantation immunosuppressive therapies have been linked to GI mucosal lesions as well. These lesions carry a poor prognostic factor disrupting the function of the GI tract, which in turn affects the pharmacokinetics of the immunosuppressive drugs eventually leading to poor graft survival and increased mortality. Mycophenolate mofetil is one of the agents more associated with intestinal erosions.

Recognizing uremic gastropathy and intervening early helps prevent post-transplant GI complications. Acid controlling therapies can be an effective prophylaxis against both gastropathies. Using enteric-coated formulation for immunosuppressive agents may slow down the mucosal insult. Treatment of H. pylori in both patient populations may help prevent further mucosal injury. Lastly, timely screening for symptoms may help start treatment early and prevent progression to serious gastropathy.

## Introduction and background

Uremic patients with chronic kidney disease (CKD) and end-stage renal disease (ESRD) as well as renal transplant recipients have been historically susceptible to gastrointestinal (GI) mucosal lesions [1,2]. GI discomfort and pain are common presenting symptoms that can put a significant toll on patient’s quality of life. While etiologies of uremic gastropathy and post-renal transplant gastropathy are different, the conditions affect a similar group of patients and are worth discussing together. A PubMed search of “uremic gastropathy” publications from 1995 to 2020 resulted in 26 articles with only 20 of them relevant to the topic in question. A similar search was done with “renal transplant gastropathy” over the same time period, resulting in 180 publications with only 10 of them relevant to the topic in question. The selected publications were analyzed in order to better understand the pathophysiology and management options of both uremic gastropathy and gastropathy induced by post-renal transplant immunosuppressive therapy.

## Review

Both uremia commonly seen in CKD and ESRD patients and treatment that generally follows renal transplant have been associated with GI complications [1,2]. The goal of this review is to focus on the difference in etiologies and management options as well as help prevent the overlap in the two gastropathies by timely intervention.

Uremic gastropathy

Upper GI complaints are common in patients with uremia [3,4]. Higher levels of urea have been linked to patients’ colonization with Helicobacter pylori and gastric mucosal inflammation. Khedmat et al. compared H. pylori infection and endoscopic findings in uremic patients with controls [3]. They observed a higher number of gastric and duodenal mucosal lesions as well as H. pylori infection in patients with uremia when compared to those with normal renal function. They concluded that it was likely due to higher serum urea levels, chronic anemia, and fluctuations in the gastric blood supply in patients on hemodialysis.

The reason for higher incidence of H. pylori infections in patients with CKD and ESRD is multifactorial. The integrity of the tight junction between the epithelial cell lining the GI tract is very important for prevention of pathogen or toxin entry [5]. Patients with CKD and ESRD are depleted of proteins claudin-1, occludin, and ZO1 that make up the tight junctions. Disruption of tight junction formation compromises integrity of the intestinal barrier leading to local and systemic inflammation, which subsequently contributes to pathogenesis of anemia, malnutrition, and cardiovascular disease.

Gastric acidity in patients with CKD can range from low to high [6]. Patients with CKD without H. pylori infection are likely to have high gastric acidity without hypergastrinemia. CKD patients with H. pylori have decreased acidity due to neutralization of acid by ammonia and overall gastric atrophy, which consequently leads to feedback hypergastrinemia that further damages gastric epithelium. As a result, H. pylori infection indirectly controls acidity of the stomach as well as fasting gastrin levels in patients with CKD. Patients with early stages of CKD are more likely to be infected with H. pylori when compared to those with advanced CKD. This is likely because angiodysplasia and erosive gastritis are more common in advanced CKD and ESRD, which may affect the ability of H. pylori to proliferate.

Metabolic acidosis is a common sequalae of CKD and is usually caused by accumulation of phosphorus and sulfate as a result of inability of kidney to alkalinize urine post-prandially [7]. Metabolic acidosis puts patients at risk for morbidity and mortality. Undergoing dialysis, however, oftentimes causes metabolic alkalosis, which subsequently decreased acidity of the stomach, putting patients at increased risk of H. pylori infection.

The exact pathogenesis of peptic ulcer disease (PUD) that subsequently develops in uremic patients with H. pylori infection remains unclear. Tseng et al. evaluated the long-term effect of H. pylori treatment in uremic patients versus non-uremic patients with PUD [8]. They studied these two patient groups over the course of six years: 34 patients with ESRD and with H. pylori infection and 67 non-uremic patients with PUD and H. pylori infection. Both groups received triple therapy to eradicate the infection. They observed that recurrence of PUD was more common in the ESRD group than that in the non-uremic PUD group. Given toxicity of bismuth preparations in uremic patients as well as in an attempt to eliminate variables, Seyyedmajidi et al. assessed correlation between creatinine clearance and eradication of H. pylori in patients with renal impairment [1]. They found no difference in H. pylori eradication between uremic and non-uremic patients. As a result, it can be concluded that there are likely factors other than H. pylori that may contribute to PUD recurrence in uremic patients [8].

Nardone et al. investigated whether elevated levels of urea in patients with CKD and ESRD could interfere with the diagnostic efficacy of the urea breath test for H. pylori infection [9]. They established that there was no difference in accuracy of the test in the setting of uremia. Moreover, the urea breath test appears to be the most sensitive diagnostic test for H. pylori infection in patients with CKD and ESRD [10,11]. Comparison of urea breath test, serology, and stool antigen testing in patients with CKD and those with normal renal function is presented in Table 1.

**Table 1:** Comparison of three diagnostic tests for Helicobacter pylori in advanced chronic kidney disease (CKD) and normal kidney function

Diagnostic test	Kidney function	Sensitivity (%)	Specificity (%)	Positive predictive value (%)	Negative predictive value (%)
Urea breath test	CKD	94	96	94	96
	Normal	95.6	100	100	95.7
Serology	CKD	86	100	100	91
	Normal	91.3	55.6	67.7	86.2
Stool antigen	CKD	58	96	91	76
	Normal	73.9	86.7	85	76.5

One of the feared PUD complications is bleeding. ESRD dialysis patients with PUD bleeding are prone to rebleeding compared to those not on dialysis and should be managed as a high-risk population [12]. The rebleeding risk tends to decrease after the first year and stabilizes after the fifth year [13]. Tseng et al. evaluated the efficacy of intravenous proton pump inhibitors (PPIs) in treating PUD in uremic patients [14]. They concluded that intravenous PPIs can protect against early rebleeding in uremic patients with PUD but are unlikely to help past seven days of treatment. Refractory PUD bleeding among ESRD patients on hemodialysis was previously reported in a number of clinical cases, where patients did not respond to traditional ulcer treatments and correction of uremia but were treated successfully with a recombinant factor VIIa [15-17]. Kario et al. further investigated these findings by measuring plasma activated factor VIIa levels in uremic patients versus healthy controls [18]. They noted that a marked increase in factor VIIa correlates to worsening renal function. They concluded that the enhanced conversion of factor VII zymogen to factor VIIa was likely related to endothelial cell injury and uremia.

Aside from previously discussed PUD affecting patients with uremia, there have been a few diseases caused by uremia that may present as PUD symptomatically. Calcific uremic arteriolopathy is not uncommon in ESRD patient population [19]. It can also present with GI symptoms if calcific uremic arteriolopathy is affecting viscera and causing Dieulafoy lesions. Lastly, uremia has also been linked to autonomic nervous system dysfunction, which some patients have described as epigastric discomfort [20]. Although both of these conditions are very different from uremic gastropathy, their symptoms are similar in presentation and can sometimes mimic one another.

Post-renal transplant gastropathy

GI discomfort is a common symptom following renal transplantation and can be caused by various immunosuppressive medications [15,21]. While taking a toll on quality of life, GI symptoms can eventually lead to poor graft survival and increased mortality [15]. Furthermore, disruption within the GI tract function may also affect the pharmacokinetics of the immunosuppressive drugs. Calcineurin inhibitors, mTor inhibitors, and corticosteroids are metabolized by the intestinal cytochrome P450 (CYP3A). Mycophenolate is partially metabolized in the stomach and small intestine and is cleared hepatically. As a result, GI disturbances can lead to a modified exposure to immunosuppressive therapy.

Patients who have undergone renal transplantation are subject to lifelong immunosuppressive therapy, which predisposes them to several GI disorders [22]. Dyspepsia is one of the more common presentations in this patient population.

Acid controlling therapies can be used to prevent dyspepsia in post-transplant recipients. Rouse et al. suggested that there is low incidence of ulcers and upper GI bleeding post-renal transplant with histamine-2 receptor antagonists (H2RA) and PPIs. They also noted similar rates of adverse events when comparing H2RA to PPIs in prophylaxis against gastropathy [23].

Immunocompromised state of renal transplant recipients makes them prone to viral and bacterial disease. Khameneh et al. investigated seroprevalence of H. pylori infection among kidney transplant recipients. They found that frequency of H. pylori infection is 47.3%, which is similar to that of the general population, as opposed to almost 60% in uremic patients [24].

Telkes et al. reviewed endoscopic data of 135 renal transplant patients in an attempt to have a better understanding of this common symptom [25]. They found that endoscopic findings were clinically significant in 84% of cases. Findings included inflammation in 46% of cases, esophagitis in 24.7% of cases, ulcer in 16.9% of cases, and erosions in 14.8% of cases. These findings were more prominent and more frequent in the first three months post-transplant. They found that use of mycophenolate mofetil increased risk of gastric or intestinal erosions by 1.8-fold [25,26].

Mycophenolate mofetil has two formulations. One is immediate-release that is absorbed in the stomach and small intestine and one is enteric-coated, which delays mycophenolate release until the small intestine [27]. Enteric-coated formulation may be associated with improved gastric toxicity. There has been a case reported of severe gastric ulcer caused by mycophenolate mofetil, which resolved following conversion to enteric-coated formulation [28]. While systemic exposure to mycophenolate mofetil is believed to correlate with the extent of toxicity, it is unclear if it is oral formulation that is responsible for GI toxicity given its direct exposure to gut lumen [27]. This theory remains unproven and more research needs to be done to better understand the dose-limiting toxicity of mycophenolate mofetil.

Other than mycophenolate mofetil, other immunosuppressants have been linked to various types of GI derangements. Patients on tacrolimus immunosuppression were found to be more prone to duodenitis [22]. Sirolimus has been noted to cause delayed healing of gastric ulcers [29].

Cocchiara et al. investigated upper GI tract hemorrhagic complications and possible recurrence of PUD post-transplantation over the course of eight years in 61 patients with ESRD [30]. All of the candidates underwent esophagogastroduodenoscopy to detect H. pylori infection. The 32 cases with H. pylori infection were divided into two groups of patients: 17 underwent treatment to eradicate the infection and 15 were left untreated. They observed that gastric or duodenal ulcers were significantly higher in patients who had the infection but were not treated when compared to those who had the infection but received treatment (5 vs 1; p=0.05) and also when compared to those without the infection (5 vs 0; p=0.05). They concluded that patients with H. pylori infection should be treated to avoid long-term complications from gastric or duodenal PUD subsequent to renal transplantation.

## Conclusions

This review sheds light on two types of gastropathies in patients with CKD and ESRD: uremic gastropathy and post-renal transplant gastropathy. Although one may think these etiologies are closely related, their etiologies and presentations are diverse. Uremic gastropathy in CKD and ESRD patients pre-transplant usually develops because of chronic uremia, anemia, and fluctuation in gastric blood supply during hemodialysis. Uremic gastropathy can also be mimicked by arteriolopathy and autonomic nervous system dysfunction caused by uremia; therefore, it is important to differentiate between them. Gastropathy in post-transplant patients tends to be associated with immunosuppressive therapy. GI lesions caused by immunosuppressive therapy disrupt intestinal function and as a result affect absorption and pharmacokinetics of immunosuppression eventually leading to poor graft survival and increased mortality. Recognizing uremic gastropathy and intervening early helps prevent post-transplant GI complications. Acid controlling therapies can be an effective prophylaxis against both gastropathies. Using enteric-coated formulation for immunosuppressive agents may slow down the mucosal insult. Treatment of H. pylori in both patient populations may help prevent further mucosal injury. Lastly, timely screening for symptoms may help start treatment early and prevent progression to serious gastropathy. 
